# The Electro-Therapeutics of Displacements of the Uterus

**Published:** 1880-07

**Authors:** Edward C. Mann

**Affiliations:** Superintendent Sunnyside Medical Retreat for Nervous Invalids; Member New York Neurological Society, Fort Washington; Member New York Medico-Legal Society, etc., New York City


ARTICLE III. PART II.

BY EDWARD C. MANN, M. D.
Superintendent Sunnyside Medical Retreat for Nervous Invalids; Member New York Neurological Society, Fort Washington; Member New York Medico-Legal Society, etc., New York City.

We come now to the consideration of the second subject of our paper, namely:

### THE ELECTRO-THERAPEUTICS OF DISPLACEMENTS OF THE UTERUS.

It has long been and is at the present day, one of the most difficult problems relating to Gynecology, how to establish a complete cure for displacement of the uterus; and it remains to be seen, whether, in many cases, the use of electricity may not prove one of the most efficient means in the hands of the profession for accomplishing the desired end. We take the liberty of referring to the normal anatomy of the supports of the uterus, which demand an especial attention, as being the parts that we wish to bring electricity to bear upon, when that organ is in a state of displacement. The uterus is suspended in the pelvis and prevented from descent by their agencies, viz.: the vaginal walls which have for their foundation the sphincta vaginal muscle; 2d. Aveolar tissue which surrounds it and binds it to the bladder, the rectum and the pelvic walls; and 3d. The ligaments which are attached to the cervix and fundus of the uterus. Retroversion is prevented by the round ligaments which are continuous with the tissue of the uterus, with which their structure is identical and which pass from the fundus to the pelvis. The Transversalis muscle also sends muscular fibres to these ligaments, which ĵact is interesting as connected with the application of electricity to the abdominal region. Anteversion is presented by the bladder, the broad ligaments and the vaginal columns. It is evident that in displacements of the uterus the uterine ligaments must be on a constant stretch, and that although naturally elastic, yet such a prolonged strain must cause them to lose in time their elasticity, and that all means employed for a permanent cure will be worthless, if we cannot in some way impart the wanting tonicity, to the partially paralyzed muscular fibres, both of the ligaments and vaginal columns. In many cases the vaginal walls and sphincta vaginæ have lost all their tonicity and are in a state of atony and semi-paralyzation, and the uterus descends for the want of its chief support.

We find in the state existing after parturition a fruitful cause for prolapsus, and a wide field for electro-therapeutics. The uterus is much heavier than usual and the vagina and sphincta vagina are very much relaxed and feeble, and the ligaments are also stretched very much. In cases of prolapsus from the above mentioned pre-disposing causes. We may hope for complete cure from the use of local and general electrization, and the beneficial action which we can exert upon the uterus and its ligaments, and upon the vagina, cannot be surpassed by any other remedial agent. We find in these cases a want of muscular contractility in all of the uterine supports. Now, if we can by faradization or galvanization, or both combined, produce contractions of the muscular fibres of the ligaments, we shall thus restore their elasticity and mechanically draw the uterus back to its normal axis ; and if we restore the tonicity of the vaginal walls and cause contraction of the longitudinal muscular fibres of the vagina, and these applications are continued for a sufficient length of time, the uterus must be of necessity gradually restored to its proper position in the pelvis. The use of pessaries affords but a temporary relief, and it remains for electro-therapeutics to establish a permament cure for this condition which, unhappily, afflicts so large a number of the female population. We have treated, successfully, four cases of prolapsus in the first degree, owing to the above mentioned causes. Under local faradization the vaginal walls and sphincta nerved their normal elasticity and tone, and ligaments were strengthened, thus mechanically restoring the uterus to its normal axis in the pelvis.

In anteversion, depending upon laxity of the abdominal walls, that takes away the support of the viscera, and which is often produced by the pernicious habit so common among our American women, of wearing very tight clothing, which forces down the viscera upon the fundus, we find the utero-vesical ligaments in a lax state, and if we wish to accomplish a permanent cure, we must restore their normal elasticity, and for this there are no means equal to electricity. In anteflexion due to an undeveloped state of the uterine parenchyma, which is a condition frequently met with, the application of electricity would stimulate the growth of the uterine tissue, and impart tonicity to the utero-vesical ligaments, and thus, perhaps, restore the uterus to its normal condition, although we should not expect such good results as in anteversion.

In retroversion due to sub-involution and relaxed support, or occurring as a sequence to parturition; we have had the best results from this mechanical effect of faradization upon the uterus and utero sacral ligaments. In retroflexion which is generally the result of some influence which weakens the uterine walls, the round ligaments are relaxed, and the abdominal walls are generally lax. In inversion, which is often dependent upon the relaxation and inertia subsequent to delivery, the application of electricity by the intra-uterine electrode may prove a valuable adjunct. Sub-involution may also act as a cause of retroflexion, and in such cases, the application of the induced current to the abdomen may be of great service.

Superinvolution or atrophy of the uterus due to the interference of the process of involution by inflammation, may be treated successfully in many instances by electricity. We may have a persistent amenorrhœa resulting from this state. In such cases we find the cavity of the uterus much diminished, and the application of the induced current to the hypogastric region externally and to the uterus directly, by means of the intro-uterine electrode will stimulate this growth of the organ, and at the same time general faradization or central galvanization, will, in a great majority of cases, probably relieve the nervousness, hysteria and neuralgia, which so often accompany atrophy of the uterus. One marked case of this kind was treated and very much benefited by the application of the induced current. The treatment consisted of local faradization through the uterus and ovaries; the negative pole being attached to the intro-uterine electrode and the positive being applied on the hypogastric region. The uterus increased in size and the amenorrhœa ceased entirely. General faradization was also employed and was the means of relieving a great deal of neuralgia and hysteria from which the patient had suffered very much. After the fourth application, the cavity of the uterus as measured by the probe, had increased from 2¼ to 2½ inches, and digital examination proved, conclusively, an increase in size and weight.

The patient when last seen had improved very much, and the probe showed the cavity of the uterus to measure 2⅝ inches. Menses regular in quantity and time. At the period of menstruation a strong induced current was passed, (a day or two previous,) through the uterus and ovaries for the purpose of exciting them, and with satisfactory results.

As the galvanic current has a greater chemical catalytic and electrotonic action than the faradic, and also causes greater molecular changes in the tissues, the effects of it would therefore be more powerful and lasting; and on the other hand as the superior mechanical effects of the faradic current are to be desired; also a combination of the two currents, or galvano-faradization, would doubtless be more efficacious in many cases than either alone, in the treatment of displacements. Central galvanization and general faradization, in many cases cannot fail to be of service in improving nutrition and relieving many of the nervous symptoms which are co-existent with displacements of the uterine.

In closing, we desire to present a case of prolapsus of long standing, which was complicated by severe coccyodynia, which was successfully cured by faradization.

Mrs. H., married, has two children; after the birth of the first child she had complete procedentia which was relieved, however, although at times she was troubled by partial descent of the uterus. She again became pregnant and after the birth of the second child she suffered from prolapsus in the second degree. This condition was complicated by severe coccyodynia, and obstinate constipation, with dyspepsia and amenorrhœa. The abdominal walls were relaxed and feeble, and physical examination showed a loss of tonicity in the vaginal walls, and the uterus prolapsed in the second degree, with a soft patulous os from which exuded a stringy mass of tenacious mucous. She reported that her last labor was prolonged and painful, and that delivery was finally accomplished by forceps. She complained of great tenderness and pain in the region of the coccyx, and could not bear pressure on it. The pain was of a sharp, neuralgic nature, and was increased by rising from a sitting posture and in defecation; application of the induced current were made about three times a week for the space of six months, being both general and local. Locally, to the vagina and uterus, and to the hypogastric and lumbar regions. Digital examination from time to time showed that the uterus was being slowly but surely restored to its normal axis. The laxity of the abdominal walls which had been very marked, decreased, and the muscles regained their tonicity under the stimulus so constantly applied. The nutrition was greatly impaired, but with the gradual restoration of the uterus and the use of ferruginous tonics came restored appetite, and cessation of the nervous irritability and prostration, which had been very marked. The uterus was finally restored to its normal axis and the menses re-appeared, owing to the long continued application by the intra-uterine electrode, which acted as a powerful local stimulus to the uterus and ovaries. The coccyodynia was treated by local applications of the induced current, the negative pole being placed over the most painful spot and the positive pole being applied on either side. These applications gave almost immediate relief, which was rendered permanent by continuing this treatment as long as the patient remained under our care. This is the only case of coccyodynia that we have treated, but the marked relief that was afforded, would seem to indicate the propriety of the use of electricity in such cases, inasmuch as the only radical cure heretofore has been formed either in section of the muscles or extirpation of the coccyx. It has been our aim in writing this paper to present practical rather than theoretical views of what has been and may be accomplished by electro-therapeutics; and in closing, we wish to speak of the necessity of a correct diagnosis in cases which we propose to treat by electricity, it being of the highest importance to the future of electro-therapeutics, that those who are interested in the subject at the present day should by careful and scientific procedure lay a foundation which cannot be shaken. The practice of electro-therapeutics requires the familiarity of the physician with all of the different branches of his profession, if he would avoid empiricism in its use; and indeed there is no other specialty which demands so extensive an acquaintance with all forms and phases of disease, and into which there is more danger of falling into a routine practice which is fatal to the scientific advancement of electro-therapeutics.

The value of electricity, together with the use of hot water, in the treatment of chronic uterine disease is but little appreciated by the profession, but from the results of our own practice, we can speak positively in its favor. We have, in all these cases, a condition of mal-nutrition and passive congestion and to restore the tissues to a normal condition and diminish abnormal secretion, we must excite reflex action, for as Dr. Emmet says, "It is only by exciting reflex action, that these nerves accompanying the vessel will cause contraction, and with increased action on their part, the natural tonicity will be restored." For exciting this reflex action I know of no means equal to electricity, and hot water, and the success with which I have treated cases of chronic uterine disease who have been patients at Sunnyside Retreat, where the trouble has been corporal, cervical or affecting the endometrium warrants me in attaching a great value to these two remedial agents in the therapeutics of uterine disease Of course in all these cases careful constitutional treatment is adopted. I have been enabled by the use of electricity, hot douches prolonged and the use of the fl. ext. viburnum prunifolium in ʒ dose to relieve some violent cases of dysmenorrhœa where all other treatment had previously failed.

